# Fatty Acid Profile and Unigene-Derived Simple Sequence Repeat Markers in Tung Tree (*Vernicia fordii*)

**DOI:** 10.1371/journal.pone.0105298

**Published:** 2014-08-28

**Authors:** Lin Zhang, Baoguang Jia, Xiaofeng Tan, Chandra S. Thammina, Hongxu Long, Min Liu, Shanna Wen, Xianliang Song, Heping Cao

**Affiliations:** 1 Key Laboratory of Cultivation and Protection for Non-Wood Forest Trees, Ministry of Education, Central South University of Forestry and Technology, Changsha, Hunan Province, People’s Republic of China; 2 Department of Plant Science and Landscape Architecture, University of Connecticut, Storrs, Connecticut, United States of America; 3 U.S. Department of Agriculture, Agricultural Research Service, Southern Regional Research Center, New Orleans, Louisiana, United States of America; National Institute of Plant Genome Research, India

## Abstract

Tung tree (*Vernicia fordii*) provides the sole source of tung oil widely used in industry. Lack of fatty acid composition and molecular markers hinders biochemical, genetic and breeding research. The objectives of this study were to determine fatty acid profiles and develop unigene-derived simple sequence repeat (SSR) markers in tung tree. Fatty acid profiles of 41 accessions showed that the ratio of α-eleostearic acid was increasing continuously with a parallel trend to the amount of tung oil accumulation while the ratios of other fatty acids were decreasing in different stages of the seeds and that α-eleostearic acid (18∶3) consisted of 77% of the total fatty acids in tung oil. Transcriptome sequencing identified 81,805 unigenes from tung cDNA library constructed using seed mRNA and discovered 6,366 SSRs in 5,404 unigenes. The di- and tri-nucleotide microsatellites accounted for 92% of the SSRs with AG/CT and AAG/CTT being the most abundant SSR motifs. Fifteen polymorphic genic-SSR markers were developed from 98 unigene loci tested in 41 cultivated tung accessions by agarose gel and capillary electrophoresis. Genbank database search identified 10 of them putatively coding for functional proteins. Quantitative PCR demonstrated that all 15 polymorphic SSR-associated unigenes were expressed in tung seeds and some of them were highly correlated with oil composition in the seeds. Dendrogram revealed that most of the 41 accessions were clustered according to the geographic region. These new polymorphic genic-SSR markers will facilitate future studies on genetic diversity, molecular fingerprinting, comparative genomics and genetic mapping in tung tree. The lipid profiles in the seeds of 41 tung accessions will be valuable for biochemical and breeding studies.

## Introduction

Tung tree or tung oil tree (*Vernicia fordii*) is a native woody oil plant in subtropical areas of China. This important economical tree has been grown in China for the production of tung oil or ornamental garden for centuries [1]. Tung tree was introduced to the United States in 1904 [2] and grown mainly in the Southern regions of the United States [2], [3]. Tung seeds contain 50–60% oil with about 80 mole % α-eleostearic acid (9*cis*, 11*trans*, 13*trans* octadecatrienoic acid) [4]. Tung oil is oxidized easily due to the three conjugated double bonds in eleostearic acid. Dried tung oil possesses excellent characteristics such as insulation, acid and alkali resistance and anticorrosion. Unlike other drying oils, tung oil does not darken with age and it becomes a widely used drying ingredient in paints, varnishes, coatings and finishes [5], [6]. Tung oil has also been used as a raw material to produce biodiesel [7], polyurethane and wood flour composites [8], thermosetting polymer [9] and repairing agent for self-healing epoxy coatings [10].

Major efforts have been directed at understanding the genetic control of tung oil biosynthesis. Many tung oil biosynthetic genes have been identified, including those coding for diacylglycerol acyltransferases (DGAT) [11], [12], delta-12 oleic acid desaturase (FAD2) and delta-12 fatty acid conjugase (FADX) [13], acyl-CoA binding proteins [14] and oleosins [15], [16]. The expression of some tung genes has been studied by northern blotting [11]–[13], quantitative real-time PCR (qPCR) [12], [14], [16]–[18] and western blotting [12]. A few tung proteins have been expressed in heterologous systems including *E. coli* [14], [19], [20], fungi [20]–[22] and *Arabidopsis* [11], [14]. However, selection of target genes for genetic engineering of plant oils is difficult because oil is biosynthesized by at least 10 enzymatic steps and each step is catalyzed by multiple isozymes [11], [23], [24]. Furthermore, it has been difficult to study tung oil biosynthesis at the protein level because these enzymes are mostly hydrophobic and membrane-localized proteins [19], [20].

Understanding fatty acid composition and genetic diversity among tung tree germplasm resources is essential for tung tree breeding and clonal improvement. A series of elite *V. fordii* clones were released in China in the 1980s for cultivation on the basis of field survey, collection and evaluation data [1]. However, these economically important germplasm resources were severely damaged by human errors and environmental factors over the past 20 years [1]. In recent years, the importance of *V. fordii* germplasm resources has been more widely recognized. We initiated germplasm collection in 2007. Some superior germplasm were collected from main distribution areas of *V. fordii* in China and planted at the Central South University of Forestry and Technology Germplasm Repository.

Microsatellites, also known as simple sequence repeats (SSRs) or short tandem repeats, are repeating sequences of 2–6 base pairs of DNA [25]. They are widely used as molecular markers in genetics and used for studies of gene duplication or deletion, marker assisted selection and fingerprinting [25]–[29]. Therefore, SSR markers could be powerful tools for genetic diversity evaluation, molecular fingerprinting identification, comparative genomics analysis and genetic mapping in tung tree. Tung tree SSR markers have been analyzed in two studies. In one study, authors analyzed 2,407 expressed sequence tag (EST) sequences from the database and identified 22 *V. fordii*-specific EST-SSR markers [30]. In the other study, 40 polymorphic SSR markers were identified from the *V. fordii* genomic DNA by AFLP of Sequences Containing repeats protocol [31]. Clearly, there is a need for developing more SSR markers for tung tree improvement.

Great progress has been developed in high throughput sequencing technology, i.e. Next Generation Sequencing, utilizing the Roche/454 Genome Sequencer FLX Instrument, the ABI SOLiD System and the Illumina Genome Analyzer. These new sequencing technologies not only offer fast, cost-effective and reliable approaches for the generation of large expression-data sets in both model and non-model plants with large and complex genomes [32]–[34], but also provide an opportunity to identify and develop unigene-derived genic-SSR markers [35]–[37]. These new genic-SSR markers are considered better markers than genomic SSR markers because they potentially code for functional proteins and can increase the efficiency of marker-assisted selection [38].

The objectives of this study were to evaluate fatty acid profiles and develop unigene-derived SSR markers in 41 tung tree accessions collected from five Provinces in China. Gas-chromatography (GC) analyzed fatty acid profiles in the mature and developing seeds. We utilized Illumina platform-based transcriptome sequencing of cDNA library from developing tung seeds and characterized microsatellites from the transcriptome sequences and developed 15 new polymorphic genic-SSR markers. We also analyzed the expression levels of the identified polymorphic SSR-associated unigenes in developing tung tree seeds and correlated their expression levels with oil content and fatty acid composition in the seeds. The fatty acid composition profiles and novel genic-SSR markers will be useful for biochemical and genetic research and tung tree improvement.

## Materials and Methods

### Plant Materials

Tung trees (*Vernicia fordii*, a diploid plant) were collected from Henan (HEN), Hunan (HUN), Hubei (HB), Guizhou (GZ) and Shanxi (SX) Provinces in China. Collecting the samples did not require specific permits because the trees were public-owned and the field studies did not involve protected species. These tung trees were planted at Central South University of Forestry and Technology Germplasm Repository. Vouchers of the sampled accessions were deposited in the University’s Herbarium. Forty-one cultivated accessions at 4-year old were used in this study. The voucher numbers, original locations and geographical coordinates of these 41 tung tree accessions are described in Table 1.

**Table 1:** Voucher numbers, collection locations and geographical coordinates of tung tree (*Vernicia fordii*).

No.	Voucher	Town, County, Province	Geographical coordinates
1	GZ11	Nanlong, Kaiyang, Guizhou	27° 0′27″N, 107°5′42″E
2	GZ16	Yangba, Ceheng, Guizhou	24°53′1″N, 105°49′28″E
3	GZ17	Tianma, Cengong, Guizhou	27°21′40″N, 108°43′45″E
4	GZ57	Yangba, Ceheng, Guizhou	24°53′1″N, 105°49′28″E
5	GZ59	Yuxi, Daozhen, Guizhou	28°53′6″N, 107°36′8″E
6	GZ78	Nigao, Wuchuan, Guizhou	28°41′25″N, 107°48′42″E
7	GZ111	Geyi, Taijiang, Guizhou	26°45′10″N, 108°10′42″E
8	GZ123	Changtian, Zhenfeng, Guizhou	25°33′35″N, 105°34′29″E
9	GZ131	Hexi, Zheng’an, Guizhou	28°26′43″N, 107°26′32″E
10	GZ164	Fuxing, Wangmo, Guizhou	25°9′56″N, 106°5′43″E
11	HB18	Shangjin, Yunxi, Hubei	33° 8′32″N, 110° 2′36″E
12	HB22	Fengshan, Luotian, Hubei	30°47′1″N, 115°23′46″E
13	HB23	Zengdu, Suizhou city, Hubei	31°42′58″N, 113°22′17″E
14	HB45	Zengdu, Suizhou city, Hubei	31°42′58″N, 113°22′17″E
15	HB60	Daxin, Dawu, Hubei	31°43′8″N, 114° 9′37″E
16	HB115	Changling, Guangshui, Hubei	31°30′48″N, 113°35′44″E
17	HB139	Fangjiaju, Yingshan, Hubei	30°38′28″N, 115°36′49″E
18	HB155	Wufeng, Yun, Hubei	32°49′27″N, 110°22′52″E
19	HB179	Manshui, Laifeng, Hubei	29°16′31″N, 109°16′35″E
20	HEN44	Xiping, Xixia, Henan	33°26′41″N, 111°5′56″E
21	HEN68	Tianguan, Xixia, Henan	33° 9′1″N, 111°41′3″E
22	HEN132	Tianguan, Xixia, Henan	33° 9′1″N, 111°41′3″E
23	HEN165	Xiping, Xixia, Henan	33°26′41″N, 111°5′56″E
24	HEN176	Jingangtai, Shang, Henan	31°47′25″N, 115°29′56″E
25	HEN177	Suxianshi, Shang, Henan	31°47′16″N, 115°35′9″E
26	HEN178	Shangshiqiao, Shang, Henan	31°57′12″N, 115°26′35″E
27	HUN39	Baisha, Luxi, Hunan	28°12′59″N, 110°13′16″E
28	HUN40	Yanjing, Yongshun, Hunan	29°15′18″N, 109°44′18″E
29	HUN41	Banqiao, Shaoyang, Hunan	27°10′34″N, 111°34′13″E
**30**	**HUN42**	**Kaihui, Changsha, Hunan**	**32°56′8″N, 109°42′13″E**
31	HUN62	Daping, Li, Hunan	29°39′49″N, 111°39′41″E
32	HUN109	Xiaojiatai, Yongshun, Hunan	29° 4′37″N, 110° 1′22″E
33	HUN118	Baiyang, Longshan, Hunan	29°25′9″N, 109°23′38″E
34	HUN159	Daping, Li, Hunan	29°39′49″N, 111°39′41″E
35	HUN160	Mujiangping, Fenghuang, Hunan	28° 5′9″N, 109°46′11″E
36	SX38	Qinghua, Qishan, Shanxi	34°25′32″N, 107°50′34″E
37	SX53	Taiyangling, Ningqiang, Shanxi	33° 1′35″N, 106° 0′9″E
38	SX79	Lianhuachi, Shanyang, Shanxi	33°17′25″N, 109°58′49″E
39	SX84	Caijiapo, Qishan, Shanxi	34°19′26″N, 107°36′17″E
40	SX119	Shuhe, Xunyang, Shanxi	32°56′8″N, 109°42′13″E
41	SX134	Huanglong, Shanyang, Shanxi	33°21′56″N, 109°37′21″E

GZ, HB, HEN, HUN and SX under “voucher” column refer to Guizhou, Hubei, Henan, Hunan and Shanxi Provinces. The bolded “HUN42” (accession No. 30) was used for cDNA library construction.

### Fatty Acid Analysis

Tung oil fatty acids were extracted from tung seeds and analyzed by GC using a similar method as described by Cao et al [12]. Briefly, tung seeds were dried in an oven (80–90°C), cracked, hulls were removed and the remaining seeds were made into fine powder with a grinder. Total seed oil was extracted with petroleum ether (approximately 10 ml/g), dried and weighted. Seed lipids in the oil extract were converted to methyl esters by KOH-methanol solution (10 mg oil extract in 0.5 ml of 1 M KOH and 40 ml methanol) and extracted with heptane. The organic phase containing lipids was transferred into a vial for GC analysis using a Gas Chromatograph (SHIMADZU GC-2014) equipped with a 60 m long capillary column (FUSED SILICA Capillary Column, SP 2340: 60 m×0.25 mm×0.2 µm film thickness-a non-bonded column highly effective for both high and low temperature separations of geometric isomers of fatty acid methyl esters, dioxins, carbohydrates and aromatic compounds) and a flame ionization detector (FID). The oven temperature was held initially at 50°C for 2 min. The oven temperature was increased from 50°C to 170°C at 10°C/min, held for 10 min, then increased from 170°C to 180°C at 2°C/min, held for 10 min and finally increased from 180°C to 220°C at 4°C/min, held for 10 min. The inlet and detector temperatures were held constant at 250°C and 300°C, respectively. The flow rate was 1 ml/min. The fatty acids in GC peaks were identified by retention times corresponding to those of the fatty acid methyl ester standards (Sigma, St. Louis, MO, USA).

### Genomic DNA Isolation

Genomic DNA was isolated from young leaves of the 41 *V. fordii* cultivated accessions using a DNA Isolation Kit (Tiangen Biotech, Beijing, China).

### RNA Isolation

Tung seeds from accession HUN42 were selected because its seeds contained the highest amount of seed oils among the 41 accessions and exhibited a typical lipid profile. The seeds were collected at lipid synthesis initiation phase (stage 1, 60 days after flowering, DAF), peak phase (stage 2, 120 DAF, equivalent to week 7 of the US collection [12]) and ending phase (stage 3, 165 DAF). Total RNA was extracted from the seeds using Micro-to-Midi Total RNA Purification System according to the manufacture’s protocols (Life Technologies Carlsbad, CA, USA). The quality and quantity of the purified RNA samples were characterized initially by agarose gel electrophoresis and NanoDrop ND1000 spectrophotometer (NanoDrop Technologies, Wilmington, DE, USA) and further assessed by RIN (RNA Integrity Number) and rRNA ratio using an Agilent 2100 Bioanalyzer (Santa Clara, CA, USA) as described [17].

### cDNA Library Construction

Equal amounts of total RNA from each of the three seed stages were pooled together for better coverage of seed development. Poly-A containing mRNA was purified from 2 mg of total RNA using oligo (dT) magnetic beads and fragmented into 200–500 bp pieces using divalent cations at 94°C for 5 min. The cleaved mRNA fragments were reverse transcribed into first-strand cDNA using SuperScript II reverse transcriptase and random primers (Life Technologies). After double-stranded cDNA synthesis, fragments were end repaired and A-tailed. The final cDNA library was created by purifying and enriching the above products with polymerase chain reaction (PCR).

### Unigene Assembly

The cDNA sequences were determined through a paired-end flow cell using an Illumina Solexa HiSeq 2000 Sequencing System at Beijing Genomics Institute (Shenzhen, China). The clean reads after DNA sequencing were de novo assembled using Trinity with default K-mers = 25 [39]. Contigs without ambiguous bases were obtained by conjoining the K-mers in an unambiguous path. The clean reads were mapped back to contigs using Trinity to construct unigenes with the paired-end information. This program detected contigs from the same transcript as well as the distances between these contigs. Finally, the contigs were connected with Trinity, and sequences that could not be extended on either end are defined as unigenes. The original sequencing data are available by contacting the authors.

### Microsatellite Analysis

The microsatellites were detected from the assembled unigenes using the MIcroSAtellite tool [40], [41]. The search parameters were set for detection of perfect di-, tri-, tetra-, penta- and hexa-nucleotide SSR motifs with a minimum of six, five, five, four and four repeats, respectively. The numbers of SSR unit type were compiled from all detected di-, tri-, tetra-, penta- and hexa-nucleotide SSR motifs. The frequencies of SSR motifs were compiled according to specific di-, tri-, tetra-, penta- and hexa-nucleotide sequences.

### Screening for Genic-SSR Markers

Genomic DNAs from leaves of three tung tree accessions (HUN42, GZ11 and HEN176) were used for testing PCR primers corresponding to 98 loci by agarose gel electrophoresis. PCR primer pairs were designed using Primer Premier 5.0. The parameters for primer design were set for primer length from 18 to 26 nucleotides, PCR product size from 100 to 400 bp and annealing temperature from 50°C to 60°C. The sequences of the forward and reverse primer pairs for 98 unigenes tested, the SSR repeated motifs and the amplicon sizes of PCR products are described (Table 2 and data not shown). PCR reactions were carried out in a total volume of 10 µL containing 10 ng of DNA template in 1×buffer, 2 mM of MgCl_2_, 200 µM of each dNTPs, 0.2 µM of each primer and 0.25 unit of Taq DNA polymerase (Takara, Japan). The PCR conditions were set at 95°C for 5 min, 35 cycles of 30 s at 94°C, 30 s at 56°C and 30 s at 72°C and a final extension of 10 min at 72°C on a DNA Engine thermal cycler (ABI9700, Applied Biosystems, Foster City, CA, USA). The amplification products were resolved on 2% agarose gels.

**Table 2:** PCR primers and test results for detecting monomorphism and polymorphism in tung tree (*Vernicia fordii*).

Primers	Unigene ID	Forward primer (5′-3′)	Reverse primer (5′-3′)	SSR motif	Amplicon (bp)	Morphism
1	VfUg4197	GAATCTTTACTGCTTATGCTGCT	TTGCCACATTCTTTCCCACT	(TTG)_7_	122	poly
2	VfUg6285	GAGGAAGGTAGAATCTCGCAA	AAGGAGCTATGGAGATGGGTT	(AGA)_7_	140	poly
3	VfUg7199	AGAAACCAGGGATCTGGAATT	CTGTAATGCGAATACAGTTGGA	(AAC)_7_	160	poly
4	VfUg8413	GATGCCCGACCTGATGAT	GACCTCAAAATGAAAAGGTGA	(AAAG)_5_	180	poly
5	VfUg15450	TTTCTTCTGTTGTGTCGTGTCTAC	CATCTGCTCTATGTCCATCGTT	(AGC)_7_	213	poly
6	VfUg15890	AAGAAGGGTGGCAAAAGTGT	TCCTTCTTTTCTCTATTGCCCT	(AG)_11_	318	poly
7	VfUg5986	TTTCGCCTATCAGACGACAAT	ATCCAGGACCAACAGAAATCA	(AG)_10_	219	poly
8	VfUg16384	GCCTGCGTTGTGTAATAATAGT	GAATGCGTATTTACACCCGA	(TATT)_5_	261	poly
9	VfUg25262	CAAGCCACAAAGAGTAACCAGT	CGAAAATCGAAATGGGACA	(TA)_10_	194	poly
10	VfUg31395	GAGGCTAACACCAGGAGACTT	TCAGAGTCTGCTTTGATTATGTG	(TC)_11_	136	poly
11	VfUg43685	AATGAAGAAGGTGACAAGACAGA	AATGGTTTGGCTTTGGTGAT	(TCTGCT)_5_	288	poly
12	VfUg52875	TGTAGTTTAGCTTCTCGCCGT	TTGGGTGTTGATTGAGTCTGTA	(GAC)_7_	185	poly
13	VfUg77143	TGCCTCTCCTCTACTACACTCGT	TCCTAGGCTAAGTAATTCGTCAA	(CTT)_7_	350	poly
14	VfUg78868	CATTCGTCCATAAATACCCACT	TGAGGAGAAACAACAGCCAGT	(TTG)_11_	159	poly
15	VfUg79257	TCTGCTAGGATCGTCATTCGT	CCTCTATACGACATTATTGAACCAG	(TCA)_5_(TGG)_5_	183	poly
16	VfUg15	CTCAATGGTGAATGGATTAGGT	GCACTTTGTTCTCTGTTAGTGGTT	(TGA)_6_	237	mono
17	VfUg487	TCCCTTGTCTCGTTATGGTCA	ACCGAGGTGGTAGAAATCTACATT	(AG)_6_	171	mono
18	VfUg575	GGTTTCAAATCCTTTCCTCG	TACGGACGGAGAAGGAGATT	(TCC)_5_	249	mono
19	VfUg3921	CCCTTTTGGGAAACATTCTTAG	TGTGATGTTTGGAGAATGGACT	(AT)_7_	309	mono
20	VfUg4003	AATCCAAAATGCAGCCCA	GCGTGAACAGAGAAATAGAGAACA	(CT)_8_	245	mono
21	VfUg4194	GGATTTTGGTGGGAAAGTTGTA	TAGGTTGTGGGTTATGTTGTGAA	(TG)_7_	312	mono
22	VfUg4251	CCAAAAGGCTCAAATCACCA	GGCATCCTTATCCTTCTTCCT	(CAA)_6_	251	mono
23	VfUg5532	GTAGAGTCAGGTGAATTGGAGGT	TTCTCACTGTTACATTCAAGCAC	(AG)_6_	195	mono
24	VfUg5684	ATTACACGCTTCTCAGCCAGT	GCATAATACTCTCCTGACAACGA	(CCA)_6_	280	mono
25	VfUg5863	ATGTATCTTCGCCCCTTGTT	TCCTCGACTGTATGTGCTCTATTA	(TC)_8_	353	mono
26	VfUg6466	GTGTCAAAGATTGGAGAGCATA	CCGCTAGAAACCATATACCCT	(GTG)_7_	231	mono
27	VfUg6678	CCACTTGAAGTTTATCAGAGACA	TTGGTATAATGTTTGCGGTTC	(TTTTA)_4_	151	mono
28	VfUg7308	TTTGAAACGGAATCGCAGA	ATCAGGGACTTGAAATCGGA	(TCC)_7_	159	mono
29	VfUg15023	CAACGGAAAACAGAATCTAACC	GAGTCAACACCATCCCTATCAT	(GATTT)_4_	202	mono
30	VfUg18315	CCAACACCACCATTACCTCC	ACACTCTTGACCCATCACCC	(AG)_10_	224	mono
31	VfUg24210	GAGAAACCATCTAAAACCCCAT	AGAAGGAACCAAACAGCAACA	(TTTC)_5_	291	mono
32	VfUg26028	TAAGCCATTGACGGAAACCT	CTGCTTTTCAACACTTCCTCTG	(AG)_9_	162	mono
33	VfUg26551	TTTCTGCTCCTGCCCTGTT	CCTTCCCTCCAAATCCAATC	(TTGT)_6_	130	mono
34	VfUg26592	TCGTAAAGGCAACTACACTGAT	ACGCAAATGTCGTTTTCTCC	(TGT)_7_	344	mono
35	VfUg28650	TCTGTAACTTGCTATCACGCTG	TACAGTTCTTATTTGTTCCTCCC	(TA)_9_	398	mono
36	VfUg28965	TCATCTACAATGGGCTCACC	TGCTTTTCTTATTTCAACCGA	(CTA)_6_	139	mono
37	VfUg29184	CCATACCCATTTTCAAGCC	GGTCCAGCGTGTTATTCG	(TC)_10_	348	mono
38	VfUg29639	TCATGTGGCTTGTGTTAAGGA	CAGCAATAAGAGTGGTCGGAT	(GTG)_7_	257	mono
39	VfUg30768	TTCAGTCCCTACCCAAACGA	GAAGATGCCCCTGATTTGTTAT	(CAC)_7_	207	mono
40	VfUg31013	TTTGCTCTTCAGGGGTCATT	ACCGTTGCCCATTTCCAC	(TG)_10_	223	mono
41	VfUg35224	TCTAACTTGGAAACGGGATG	ATGGGGAGATTTAGGAGGAG	(TGA)_6_	223	mono
42	VfUg36152	AGCATTGACTTTTCACTGGTTC	TAAGCATAGAGAGATGGGATTGT	(AT)_10_	273	mono
43	VfUg36575	TTTTGTCCAGTAGATGGCTTAG	GAGAATCCCAATGCTCAGTC	(TTAGT)_5_	116	mono
44	VfUg39010	TTCAGCATCCAAAACTTTACTT	ATGTTTCCCTCAGGTTATCTATT	(ATA)_6_	151	mono
45	VfUg39497	TCTGATAGATAGCGGAGCC	GTGGGTTGAGGACGAAGC	(AAAT)_5_	346	mono
46	VfUg43215	GTCACTTGGGGCATTTAGGTA	GCATTCACGCACTCAACACT	(GATTTC)_4_	195	mono
47	VfUg44021	TTCTTCTGCCTCCTCGTCCT	TGATTGGGATTGGTGCTCTG	(AAG)_7_	184	mono
48	VfUg45108	AACCCTGTTGCTGGGATACT	AATACAAGAGTTTGGCACCGA	(TTGA)_5_	170	mono
49	VfUg47336	TCCCTTTTCGCTTTTCGTG	AAACACTTCTCAGCCTCACAGC	(AG)_9_	240	mono
50	VfUg49167	ATAAACTCCTGCTGCTCCG	CTGTCCAAAACTACAAACATCAA	(GTGGCA)_4_	231	mono
51	VfUg52207	TGAAATCAGCAGAACAGAACCTC	GCCAGCCCAAATGTCCAA	(GAGAA)_4_	219	mono
52	VfUg55989	TCAGCATTCCACACCCAA	CTAGATGCCTTTCCAACCATA	(TATG)_5_	298	mono
53	VfUg56462	TTTTCGCAGTTATCACCATTG	ACAATTATGCCATCCTATGACAC	(AC)_10_	326	mono
54	VfUg77652	TGCCACTATATGAGTTTGTGTACG	GGTATCATTTGGGTCCCTGTAA	(AGT)_7_	313	mono
55	VfUg80977	TCCCCATCCTCTGATTCTGA	GCTGCCCAATCTACAAACAA	(CTC)_7_	190	mono
56	VfUg77408	CATCTGTGTCAAACGCTCCA	GAATCGGATACTTAGGTAGGGTTA	(CTG)_7_	366	mono

Vf and Ug under “unigene ID” column represent the abbreviation of tung tree (*Vernicia fordii*) and unigene followed by the unigene number.

### Development of Polymorphic Genic-SSR Markers

The loci that generated PCR products with expected sizes on agarose gel were assessed for polymorphisms by high-resolution capillary electrophoresis. PCR products were generated by Touchdown PCR with fluorescently labeled M13 (–21) (5′-TGTAAAACGACGGCCAGT-3′) sequence-tag method [42]. Touchdown PCR was carried out using the following program: 95°C for 5 min; 30 cycles of 30 s at 94°C, 45 s at 56°C and 45 s at 72°C; 10 cycles of 30 s at 94°C, 45 s at 53°C and 45 s at 72°C; and a final extension of 5 min at 72°C. Fluorescently labeled PCR products were initially evaluated by 2% agarose gel electrophoresis and then analyzed by capillary electrophoresis with the GeneScan-500 LIZ Size Standard on an ABI 3730XL sequencer and their sizes were determined with GeneMapper version 4.0 (Applied Biosystems).

### Quantitative Real-Time PCR

The expression patterns of the 15 polymorphic SSR-associated unigenes in developing tung seeds were studied by quantitative real-time PCR (qPCR) using SYBR Green method essentially as described [12]. PCR primers in Table 2 were designed to identify polymorphism by amplifying DNA fragments from genomic DNA. Therefore, new sets of primers were designed to analyze the expression levels by amplifying cDNA corresponding to the identified 15 polymorphic genes in the seeds (Table 3). Tung tree *EF1a* gene was used as the reference gene [43]. The qPCR assay was carried out with three replicates in each reaction using the Bio-Rad CFX system (Bio-Rad). Unigene specific primers are listed in Table 3. PCR was performed in a 20 µL volume containing 2 µl diluted cDNA, 250 nM each primer and 1×SYBR Premix Ex Taq II (TaKaRa). The results were analyzed using the comparative Cq method which uses an arithmetic formula, 2^−ΔΔCq^, to obtain results for relative quantification [44].

**Table 3:** PCR primers for polymorphic SSR-associated unigenes in tung tree (*Vernicia fordii*).

Unigene	Primers	Tm (°C)	GC (%)	Amplicon (bp)
VfUg4197	F:TGCCACATTCTTTCCCACT	55.8	47	173
	R:GCTGACACTGCTTCTACTGCTAT	56.3	48	
VfUg6285	F:TGCTGGGACGGTCGGTA	58.1	65	129
	R:GGAATCGCCACACGCTT	57.0	59	
VfUg7199	F:GTGATTATGGTGACTATGTGTTTG	54.5	38	115
	R:TCTTCCGCATTTGGTATTG	54.3	42	
VfUg8413	F:TGTTTGCAATCCATGCTT	50.0	38	98
	R:GTTTGCAATTGACAAATG	48.0	33	
VfUg15450	F:TCTGTGGATTCGGATTTCTTT	56.5	38	134
	R:CTGTGGTGGACCCTCTTCTC	56.4	60	
VfUg15890	F:GTGTTCTCTTGAAAGGCGA	53.2	47	204
	R:GGTGGAGGATTTGATGGC	55.2	56	
VfUg15986	F:GATTTCTGTTGGTCCTGG	49.7	50	114
	R:CCGAGTTTCACTTGGGTA	50.8	50	
VfUg16384	F:GGGGTGTTCCAACTGCTA	53.3	56	192
	R:TTGCTGGCTCATAATAAGATAA	53.3	32	
VfUg25262	F:GCCATTATTGAAGCCGT	51.0	47	108
	R:CACCCTTGAACTCGTAGC	50.5	56	
VfUg31395	F:GAGGCTAACACCAGGAGACTT	55.3	52	123
	R:TGATTATGTGGGAAAACGAGA	55.7	38	
VfUg43685	F:TTATGTGCCGCCACCTTAT	56.5	47	137
	R:CGCAGATTCCAGATGACCA	57.0	53	
VfUg52875	F:GGAAGCCAGTAATGGATGTT	54.1	45	216
	R:GCTGCCCAGAAGAATAGAAG	54.4	50	
VfUg77143	F:AGATTTTACCACCGCTTC	49.7	44	225
	R:CCTTGTAGGCATCCCATAG	52.8	53	
VfUg78868	F:TGAGGAGAAACAACAGCCAGT	57.2	48	160
	R:GCATTCGTCCATAAATACCCAC	58.9	46	
VfUg79257	F:ATTTTCAGTAGCAATCTTCCT	50.7	33	125
	R:CTTCTGGTTCAATAATGTCGT	52.1	38	

### Correlation Analysis

Gray correlation analysis software (V2.1) was used to generate correlation coefficient between gene expression levels and oil content or fatty acid composition [45]. The oil content/fatty acid composition was used as reference series and the mRNA levels of the 15 genes were used as comparison series. The higher correlation coefficient between the mRNA levels and oil content/fatty acid composition means the more positive effect of the gene product on oil content/fatty acid composition.

### Genetic and Phylogenetic Analyses

The number of alleles was detected by capillary electrophoresis of the PCR-amplified products. Genetic parameters including the number of alleles (*Na*), effective number of alleles (*Ne,* the number of alleles that would be expected in a locus in each population), expected heterozygosity (*He,* the probability that any two alleles, chosen at random from the population, are different to each other at a single locus) and observed heterozygosity (*Ho*) were estimated based on the capillary electrophoresis data with POPGENE version 1.31 [46]. Polymorphism information content (*PIC*) values at each locus were calculated as described [47], [48]. Coefficients of genetic similarity for the 41 accessions were calculated using the SIMQUAL program of NTSYS-pc Version 2.10 (Exeter Software) [49]. The 15 genic SSR markers identified in this study were used initially for the phylogenetic analyses of the 41 accessions. In addition, polymorphism has been studied by other laboratories in tung tree [31]. To expand the phylogenetic analysis of polymorphic SSR-associated genes, we also analyzed polymorphism corresponding to genomic SSRs reported in the published paper [31]. Seventeen genes were confirmed with polymorphism. The names of loci, the sequences of PCR primers and SSR motifs for the confirmation studies are presented in Table S1. Phylogenetic analysis was therefore performed using the 32 polymorphic genes including 15 genes from current studies and 17 genes confirmed from previous studies. Unweighted Pair Group Method with Arithmetic Mean (UPGMA) dendrogram was constructed based on the genetic similarity matrix with the SHAN clustering program [50].

## Results

### Fatty Acid Composition and Accumulation in Tung Tree Seeds

**Figure 1:**
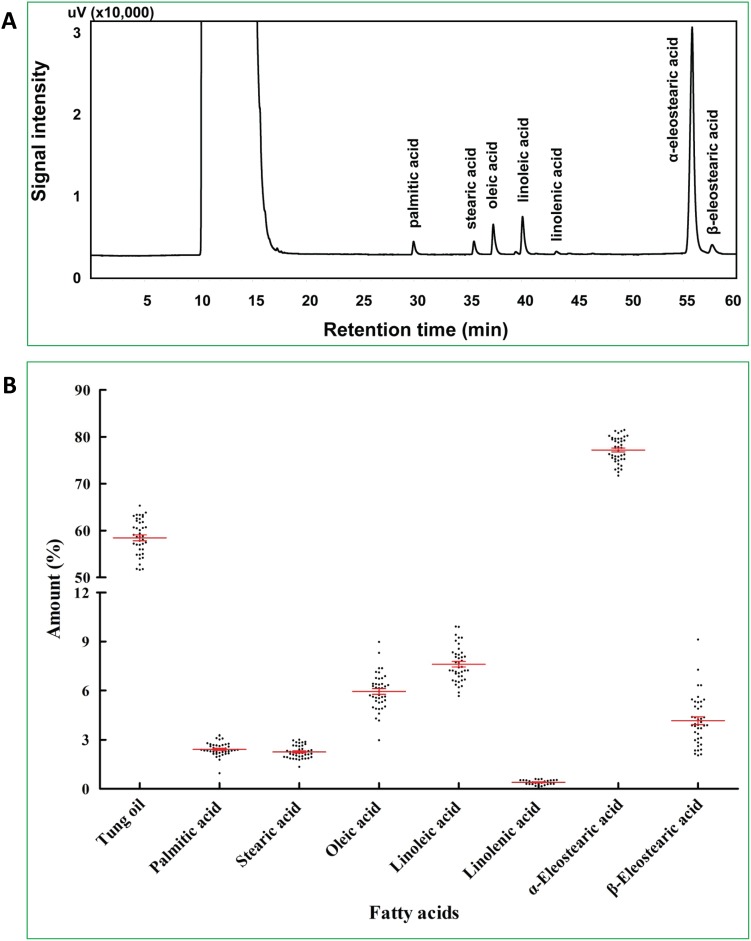
Fatty acid composition in mature seeds of tung tree. Tung oil was extracted from tung seeds by petroleum ether. Seed lipids were converted to methyl esters by KOH-methanol solution followed by separation and detection by GC-FID. (A) GC separation of fatty acids in tung oil from HUN4241 accessions. The first peak on the chromatogram was the solvent peak. (B) Fatty acid composition in mature tung seeds. The means and standard deviations of GC results from 41 accessions are marked in red color.

Forty-one cultivated tung tree accessions used in this study were collected from five Chinese Provinces, planted at Central South University of Forestry and Technology Germplasm Repository and deposited in the University’s Herbarium (Table 1). The major economical value of tung tree is the unique α-eleostearic acid (9*cis*, 11*trans*, 13*trans* octadecatrienoic acid) in tung oil extracted from the seeds. We therefore analyzed the fatty acid profiles of mature seeds from all 41 tung tree accessions. GC typically identified 7 fatty acid peaks in tung oil corresponding to palmitic acid (16∶0), stearic acid (18∶0), oleic acid (18∶1), linoleic acid (18∶2), linolenic acid (18∶3), α-eleostearic acid (18∶3) and β-eleostearic acid (Figure 1A). Alpha-eleostearic acid consisted of the great majority of tung oil with an average of 77.2% of the total fatty acids in the seed oil from the 41 accessions (Figure 1B). The relative abundances of the other 6 fatty acids from the 41 accessions were linoleic acid (7.6%), oleic acid (5.9%), β-eleostearic acid (4.2%), palmitic acid (2.4%), stearic acid (2.3%) and linolenic acid (0.4%) (Figure 1B). The amount of linolenic acid was minimal and undetectable in oils from several accessions (Figure 1B). Tung oil and fatty acid profiles show that the initiation of α-eleostearic acid accumulation started at 60 DAF and peaked at 120 DAF; whereas the amount of other fatty acids declined during seed development (Table 4).

**Table 4:** Tung oil and fatty acid accumulation in developing tung tree seeds.

Days after flowering	60 DAF	75 DAF	90 DAF	105 DAF	120 DAF	135 DAF	150 DAF	165 DAF
Tung oil (w/w)	0.23±0.02	3.58±0.47	18.02±0.98	42.92±2.79	59.78±2.87	60.12±2.54	65.78±2.91	65.32±2.01
Palmitic acid (16∶0)	22.6±1.15	13.78±1.82	8.47±0.19	6.89±0.80	5.32±0.36	3.92±0.56	2.6±0.54	2.3±0.48
Stearic acid (18∶0)	4.65±0.41	4.22±0.31	3.78±0.89	3.44±0.40	3.26±0.22	2.98±0.37	2.55±0.26	2.19±0.20
Oleic acid (18∶1)	12.64±0.64	10.19±0.51	9.98±0.77	9.62±0.72	9.08±0.32	8.48±0.44	7.06±0.19	6.4±0.17
Linoleic acid (18∶2)	40.45±2.14	32.56±2.07	12.87±1.70	10.68±0.33	8.86±0.65	7.95±0.93	7.28±0.56	7.23±0.66
Linolenic acid (18∶3)	17.96±1.63	31.4±2.57	20.78±1.47	8.71±0.77	8.67±1.67	0.64±0.06	1.00±0.03	0.28±0.02
α-Eleostearic acid (18∶3)	1.36±0.08	6.87±0.81	42.56±1.77	56.88±1.63	59.72±1.51	70.25±2.28	73.43±2.08	75.28±2.20
β-Eleostearic acid (18∶3)	0.34±0.04	0.98±0.05	1.56±0.07	3.78±0.11	5.09±0.10	5.78±0.23	6.08±0.21	6.32±0.18

DAF: Days after flowering. The means and standard deviations of three determinations are presented.

### High Quality RNA Isolation from Tung Tree Seeds

As an initial step towards the goal of improving the agronomic traits of tung tree and oil contents in the seeds, we began to characterize DNA microsatellites and develop unigene-derived SSR markers. Tung seeds from accession HUN42 were selected for cDNA library construction because its seeds contained the highest amount of tung oil. Total RNA samples were isolated from three developmental stages of tung seeds. The amount and quality of RNA preparations were assessed by Agilent 2100 Bioanalyzer to be sure that high quality RNA was used for construction of cDNA library. These RNA preparations were extremely high quality as indicated by high RNA integrity number (RIN>8) and high 28S:18S rRNA ratio (close to 2.0) in the RNA preparations (Figure S1).

### Unigene Identification from Tung Tree Seed Transcriptome

**Figure 2:**
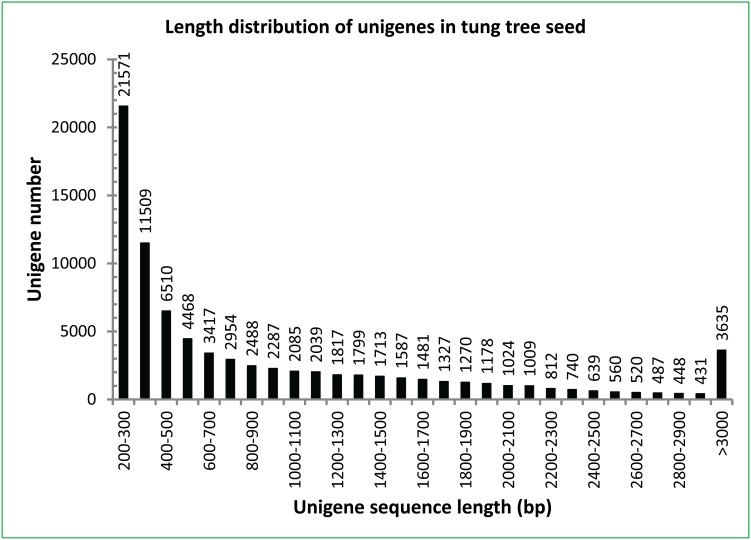
Unigene distribution from the sequenced transcriptome of tung seeds. The cDNA sequences were determined by Illumina Solexa HiSeq 2000 Sequencing System and de novo assembled using Trinity program.

The pooled RNA from the three seed stages were used to construct cDNA library for better representation of the whole seed developmental stages. Sequences of the complete cDNA library were assembled into 81,805 unigenes with a mean length of 945 bp (Figure 2). These unigenes were used to identify microsatellites and develop SSR markers.

### Types of Microsatellites in Tung Tree Unigenes

**Figure 3:**
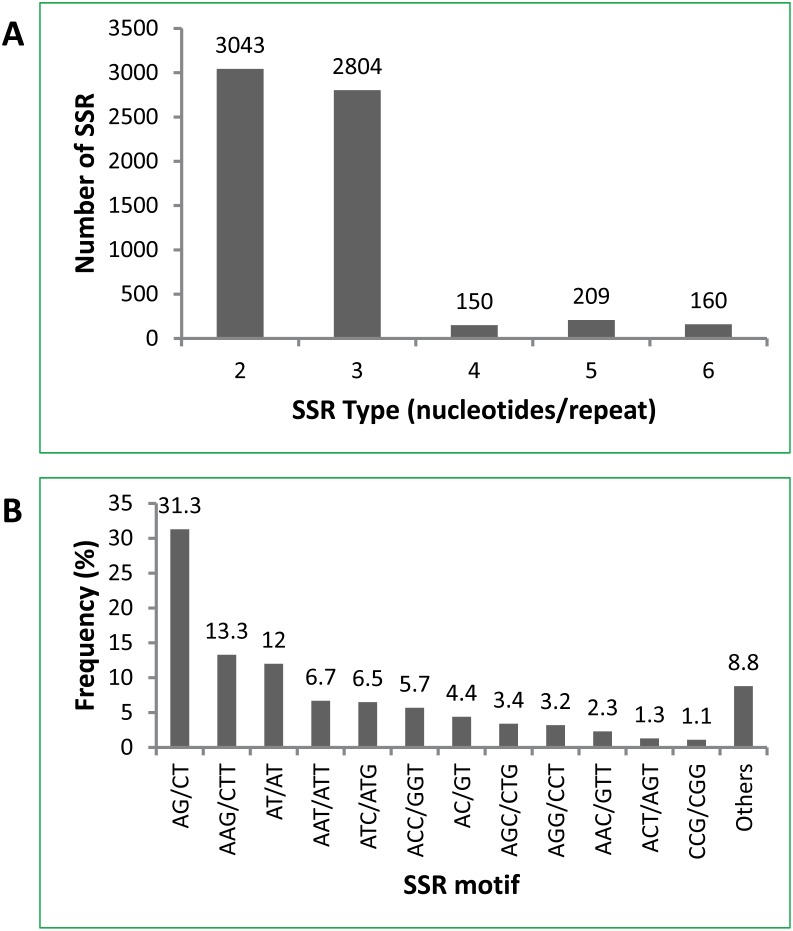
Types and frequencies of SSRs identified from the unigenes from tung seed cDNA library. The search parameters were set for detection of perfect di-, tri-, tetra-, penta- and hexa-nucleotide SSR motifs with a minimum of six, five, five, four and four repeats, respectively. (A) Distribution of SSR unit type, (B) Frequency of classified SSR motifs.

MIcroSAtellite tool was used to screen the types of microsatellites from the unigene dataset obtained from tung tree seeds. A total of 6,366 SSRs in 5,404 unigenes contained di-, tri-, tetra-, penta- or hexa-nucleotide repeats (Figure 3A). They represented 6.6% of the 81,805 unigenes in tung seeds with at least one of the considered SSR motifs. The maximum and minimum lengths of the SSR repeats were 179 and 12 nucleotides respectively, with an average length of 16 nucleotides. They were mostly di-nucleotide (47.8%) and tri-nucleotide (44.0%), and less tetra-nucleotide (2.4%), penta-nucleotide (3.3%) and hexa-nucleotide (2.5%) (Figure 3A). The complete list of 6,366 SSRs from 5,404 unigenes with di-, tri-, tetra-, penta- or hexa-nucleotide repeats is presented as “Supporting Information” (Table S2).

### Frequencies of Microsatellites in Tung Tree Unigenes

The most abundant SSR motif was (AG/CT), which accounted for 31.3% of the total SSR motif (1993 out of 6,366 potential SSRs) (Figure 3B). Other abundant SSR motifs included (AAG/CTT, 13.3%), (AT/AT, 12.0%), (AAT/ATT, 6.7%), (ATC/ATG, 6.5%), (ACC/GGT, 5.7%) and (AC/GT, 4.4%) (Figure 3B). Among the di-nucleotide repeats, the AG/CT motifs showed the most frequency (65.5%, 1993), followed by the AT/TA motifs (25.1%) and AC/GT (9.3%). Among the tri-nucleotide repeats, AAG/CTT motifs were the most common, accounting for 30.2% (847), followed by AAT/ATT (15.2%) and ATC/ATG (14.7%). Other motifs were identified in less significant numbers. The complete list of the frequency of identified SSR motifs is presented as “Supporting Information” (Table S3).

### Screening for Genic-SSR Markers by Agarose Gel Electrophoresis

**Figure 4:**
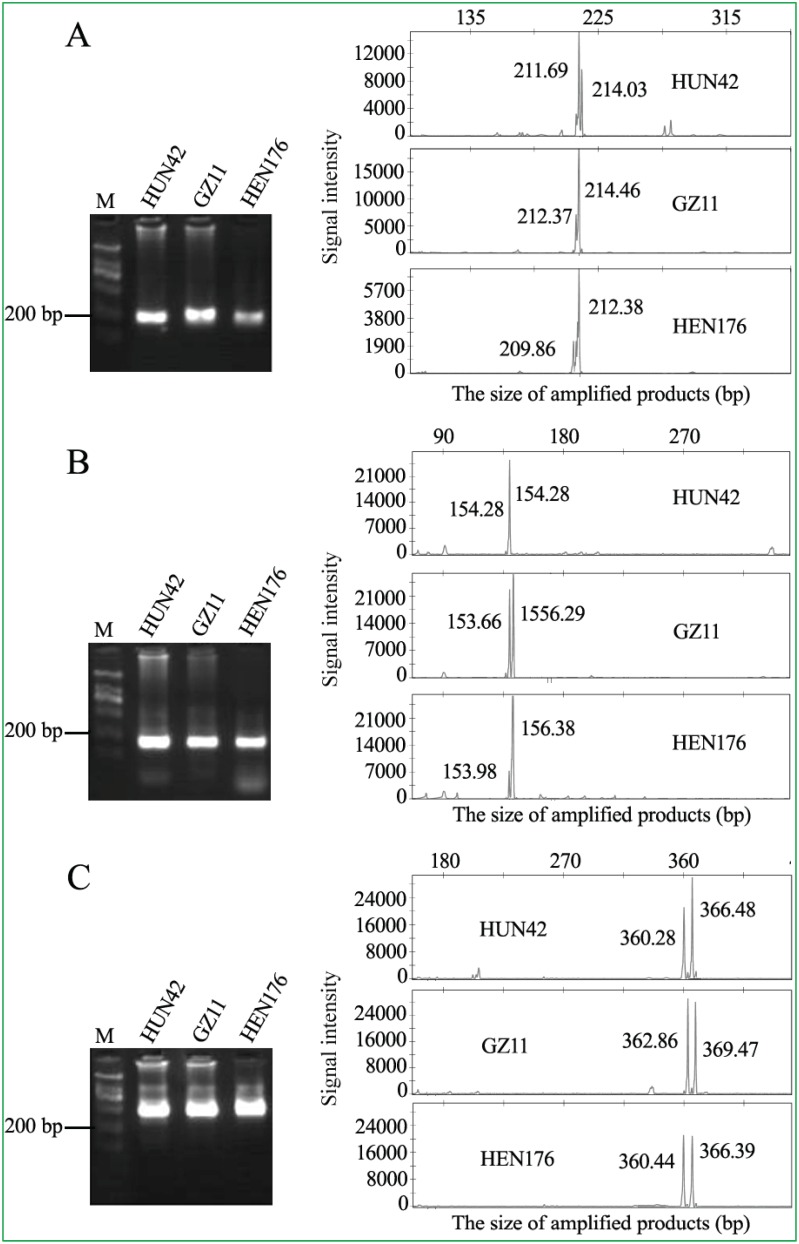
Polymorphism of genic-SSRs revealed by agarose gel and capillary electrophoresis. PCR primers for VfUg25262, VfUg3139 and VfUg77143 loci (unigenes) were used to amplify DNA fragments from genomic DNA of three tung tree accessions (HUN42, GZ11 and HEN176). The PCR products were separated by 2% agarose gel electrophoresis (left panels) and capillary electrophoresis (right panels). Vf and Ug in the locus name represent the abbreviation of tung tree (*Vernicia fordii*) and unigene. M represents the DNA size standards (DL600 DNA ladder: 100, 200, 300, 400, 500 and 600 bp). (A) VfUg25262 locus, (B) VfUg3139 locus, (C) VfUg77143 locus.

After eliminating undesirable unigenes (sequences were too short and contained unusual GC content and Tm for optimal primer design) and avoiding duplications of those published SSRs [30], [31], 98 loci were selected from the 5,404 unigenes in tung seeds for polymorphic genic-SSR development. PCR primer pairs corresponding to the 98 loci were designed using the criteria described in “Materials and Methods” (Table 2). These primers were used initially to amplify DNA fragments from genomic DNA of three tung tree accessions. Agarose gel shows that the PCR primer pairs for VfUg25262, VfUg31395 and VfUg77143 loci amplified DNA fragments with approximately 200, 150 and 350 bp, respectively, from the genomic DNA of tung tree HUN42, GZ11 and HEN176 accessions (Figure 4, left panels). Similar results from agarose gel electrophoresis revealed that 56 loci generated products of expected sizes (Table 2), whereas 27 loci yielded nonspecific PCR products and 15 loci yielded no PCR products (data not shown).

### Development of Polymorphic Genic-SSRs by Capillary Electrophoresis

Capillary electrophoresis is more accurate to estimate the sizes of DNA molecules than agarose gel electrophoresis. The positively identified 56 loci by agarose gel electrophoresis were used for polymorphism analysis by capillary electrophoresis. Figure 4 (right panels) clearly shows that capillary electrophoresis separated each band shown on agarose gel (left panels) into two DNA fragments with minor size differences (right panels).

**Figure 5:**
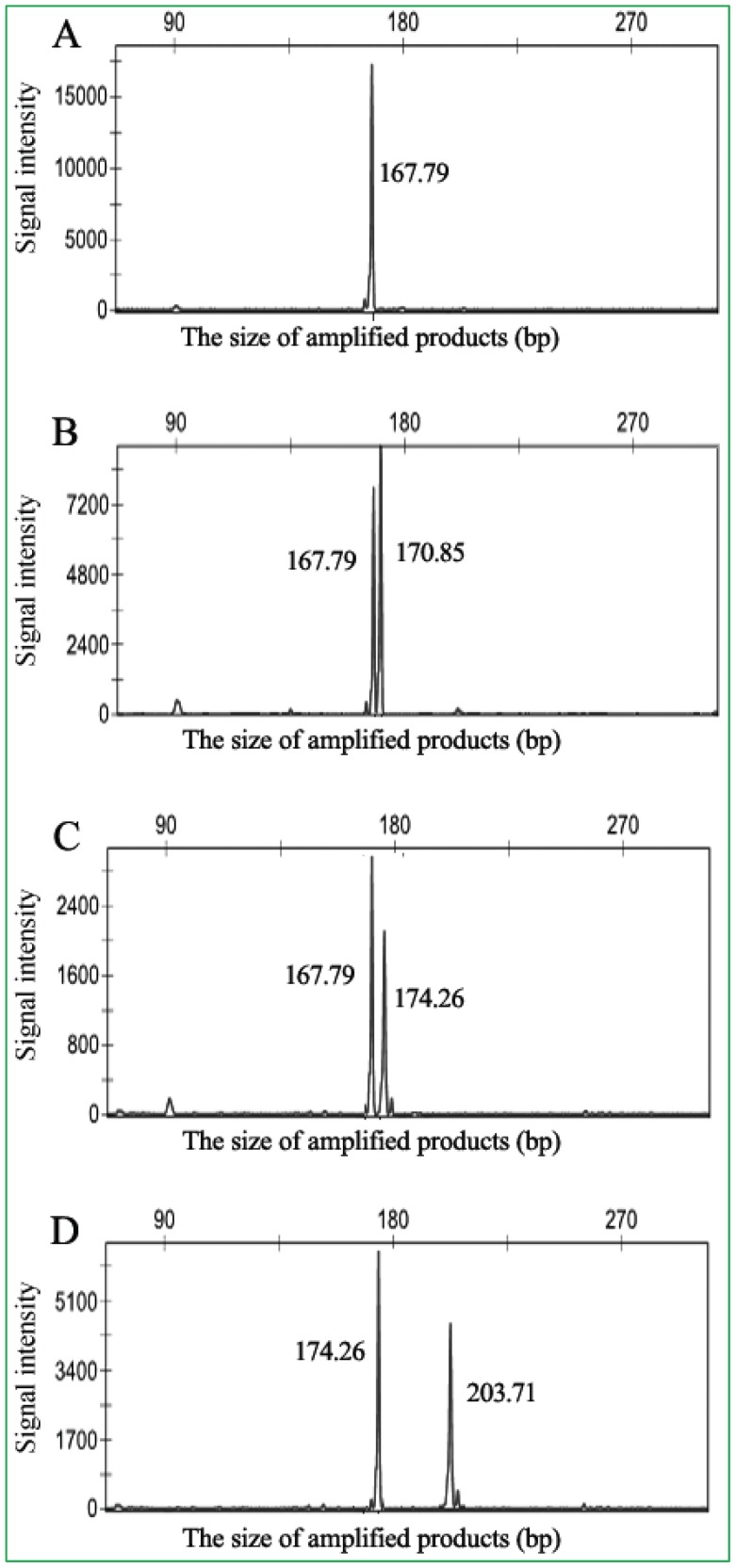
Identification of the number and size of polymorphic genic-SSRs by capillary electrophoresis. The results from VfUg78868 (polymorphic gene) are shown as an example. PCR primers for VfUg78868 locus were used to amplify DNA fragments from genomic DNA of 41 tung tree accessions. The results from four accessions representing the complete set of 4 alleles are presented. The PCR products were separated by capillary electrophoresis. The length of the PCR product on the figure is 18 bp longer than the actual size in Table 3 because an 18 bp fluorescent primer M13 (–21) (5′-TGTAAAACGACGGCCAGT-3′) was used for labeling PCR products. (A) accession GZ131, (B) accession HEN176, (C) accession HUN42, (D) accession HB60.

Figure 5 shows an example of using PCR primers for VfUg78868 locus to analyze the numbers and the sizes of this polymorphic SSR-associated gene in 4 tree accessions. PCR assay for VfUg78868 locus amplified a 168 bp fragment from accession GZ131, suggesting a homozygous gene in this accession (Figure 5A). Two different DNA fragments (heterozygous gene) from accession HEN176 (168 and 171 bp), accession HUN42 (168 and 174 bp) and accession HB60 (174 and 204 bp) were detected by this method (Figure 5B–D). The four sizes of PCR fragments separated by capillary electrophoresis (168, 171, 174 and 204 bp) indicated that there were four alleles of VfUg78868 locus in the four tree accessions (Figure 5). This method demonstrated that 41 out of the 56 loci exhibited monomorphism and 15 loci displayed polymorphism among the three tested accessions (Table 2). These 15 genic-SSR markers were validated by capillary electrophoresis using genomic DNA from all 41 *V. fordii* accessions (Table 5). The number and sizes of all alleles of the 15 loci detected among the 41 tree accessions by capillary electrophoresis are summarized in Table 5. The 15 unigene sequences have been deposited in the GenBank database under the accession numbers shown in Table 5.

**Table 5:** GenBank number, genetics parameter, allele size and putative function of 15 polymorphic genic-SSRs developed from 41 tung tree accessions.

ID	Locus	GenBank no.	*N_a_*	*N_e_*	*H*_o_	*H_e_*	*PIC*	Allele size (bp)	Putative function	GenBank reference
1	VfUg4197	KC991187	2	1.71	0.05	0.42	0.33	118, 121	conserved hypotheticalprotein	ref|XP_002329124.1|
2	VfUg6285	KC991188	2	1.30	0.27	0.24	0.21	122, 137	RNA splicingprotein mrs2	ref|XP_002530669.1|
3	VfUg7199	KC991189	4	1.16	0.15	0.14	0.13	157, 160,172, 178	conserved hypotheticalprotein	ref|XP_002533815.1|
4	VfUg8413	KC991190	2	1.19	0.17	0.16	0.14	178, 186	no hit	
5	VfUg15450	KC991191	2	1.19	0.07	0.16	0.14	177, 213	transcription factorVIP1-like	gb|ABK96202.1|
6	VfUg15890	KC991192	4	1.55	0.34	0.36	0.34	294, 304,334, 336	putative phosphate-inducedprotein	ref|XP_002298705.1|
7	VfUg15986	KC991193	3	2.07	0.66	0.52	0.46	327, 329, 331	anthocyanidin reductase	ref|XP_002305639.1|
8	VfUg16384	KC991194	5	1.96	0.29	0.50	0.45	278, 282, 286,290, 294	V-type proton ATPasesubunit H-like	gb|EOY10174.1|
9	VfUg25262	KC991195	4	2.62	1.00	0.63	0.54	192, 194, 196, 198	3′-N-debenzoyl-2′-deoxytaxolN-benzoyltransferase	ref|XP_002533002.1|
10	VfUg31395	KC991196	2	1.33	0.29	0.25	0.22	136, 138	no hit	
11	VfUg43685	KC991197	10	2.80	0.76	0.65	0.62	310, 317, 320, 325,355, 375, 380,385, 390, 395	plant cadmiumresistance 10-likeisoform 1	ref|XP_002518818.1|
12	VfUg52875	KC991198	8	2.20	0.59	0.55	0.53	361, 367, 370, 373,376, 379, 382, 385	NifU-like protein 4	ref|XP_002524990.1|
13	VfUg77143	KC991199	4	3.16	1.00	0.69	0.62	342, 345, 348, 351	disease resistanceprotein RPM1	ref|XP_002527910.1|
14	VfUg78868	KC991200	4	2.57	0.56	0.62	0.55	150, 153, 156, 186	protein binding protein	ref|XP_002518472.1|
15	VfUg79257	KC991201	4	1.63	0.46	0.39	0.36	179, 184, 189, 194	hypothetical proteinRCOM_1466500	ref|XP_002514576.1|
Mean			4	1.90	0.44	0.42	0.38			

Vf and Ug under “locus” column refer to tung tree (*Vernicia fordii*) and unigene, respectively.

*N_a_*, number of alleles; *N_e_*, effective number of alleles; *H_o_*, observed heterozygosity; *H_e_*, expected heterozygosity. *PIC*, polymorphism information content.

### Functional Annotation of Polymorphic SSR-associated Unigenes

GenBank database search was used to uncover the potential functions of the 15 polymorphic SSR-associated unigenes. The 15 unigene sequences were blasted against the GenBank nonredundant database using BLASTX with an E-value <1×10^−5^. Thirteen of the 15 sequences showed significant similarities to known genes (Table 5). Ten of the 15 loci putatively coded for a variety of proteins including RNA splicing protein mrs2, transcription factor VIP1-like protein, phosphate-induced protein, anthocyanidin reductase, V-type proton ATPase subunit H-like protein, 3′-N-debenzoyl-2′-deoxytaxol N-benzoyltransferase, plant cadmium resistance 10-like isoform 1, NifU-like protein 4, disease resistance protein RPM1 and protein binding protein (Table 5).

### Polymorphic Evaluation of the Genic-SSRs

Genetic analysis estimated that the number of alleles (*N_a_*) per locus ranged from two to ten, the expected heterozygosity (*H_e_*) per locus ranged from 0.140 to 0.692 and the polymorphism information content (*PIC*) per locus ranged from 0.134 to 0.624 (Table 5). Five of the 15 loci (VfUg25262, VfUg52875, VfUg78868, VfUg77143 and VfUg43685) were high polymorphic (*PIC*>0.5) and five (VfUg4197, VfUg15890, VfUg79257, VfUg16384 and VfUg15986) were moderate polymorphic (0.25<*PIC*<0.5) (Table 5).

### Gene Expression and Correlation with Seed Oil Content and Fatty Acid Composition

**Figure 6:**
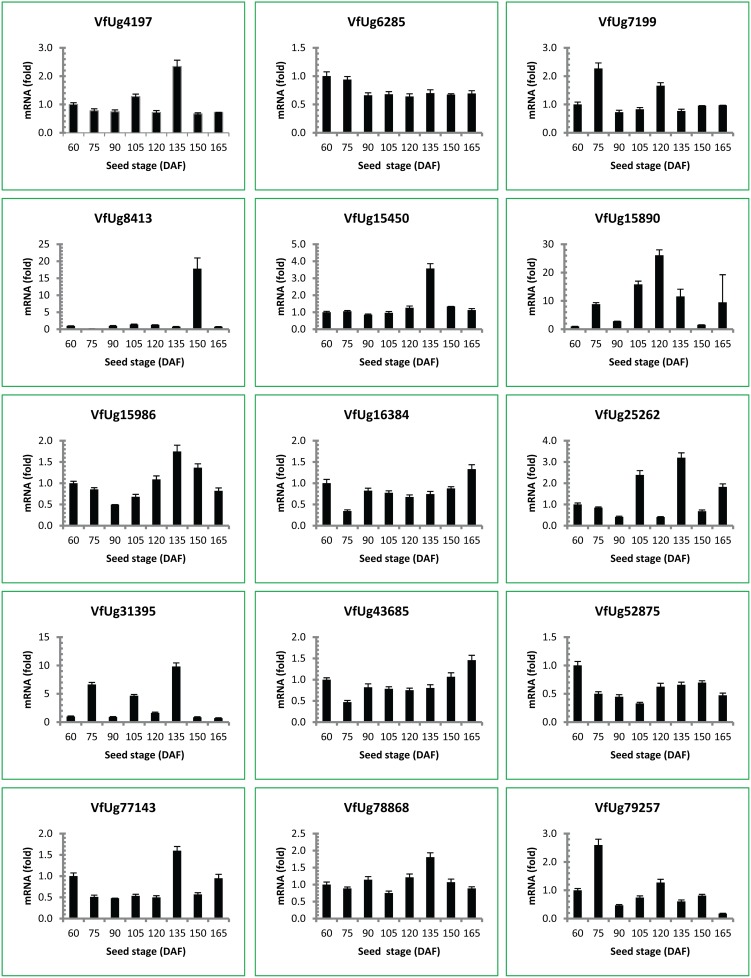
Expression profiles of the polymorphic SSR-associated unigenes in tung tree seeds. The mRNA levels were quantified by qPCR using total RNA from eight seed developmental stages. The relative abundance of mRNA levels at 60 DAF was set at 1.0. qPCR was performed in triplicates by SYBR Green qPCR assay using *EF1A* gene as the reference gene. The mean and SD from triplicates are presented in the figure.

**Figure 7:**
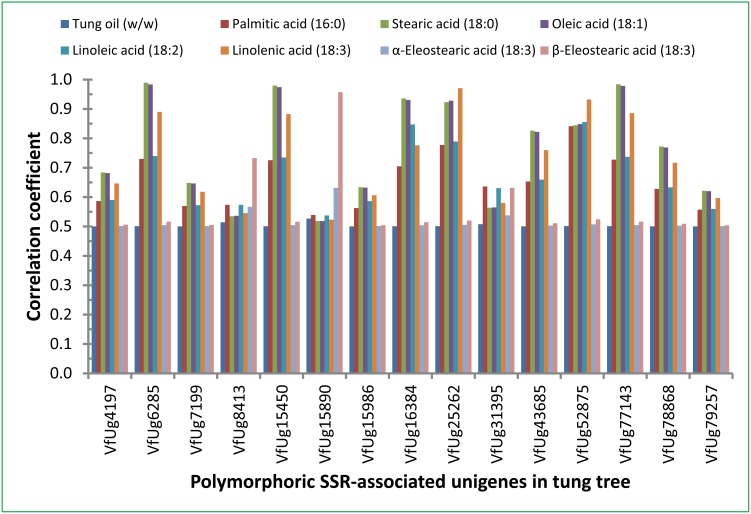
Correlation between expression levels of polymorphic SSR-associated unigenes and oil and fatty acid composition in tung seeds. Gray correlation analysis was performed to generate correlation coefficient between gene expression levels and oil content and fatty acid composition. The higher correlation coefficient between the mRNA levels and oil content/fatty acid composition means the more positive effect of the gene product on oil content/fatty acid composition.

Quantitative real-time PCR was used to study the expression of 15 polymorphic SSR-associated unigenes during tung seed development. Expression of these genes was experimentally confirmed by qPCR using RNA isolated from eight seed development stages (Figure 6). The expression levels of some genes were increased during seed development including VfUg4197, VfUg8413, VfUg15450, VfUg15890 and VfUg15986. The gray correlation analysis software evaluated the relevance between the mRNA levels of these genes and oil content/fatty acid composition (Figure 7). There was not significant correlation between the expression levels and oil content or α-eleostearic acid, the major component of tung oil (Figure 7). However, a strong correlation was obtained between mRNA levels of some genes (VfUg6285, VfUg15450, VfUg16384, VfUg25262, VfUg52875 and VfUg77143) and fatty acid composition (palmitic acid, stearic acid, oleic acid, linoleic acid and linolenic acid) (Figure 7).

### Phylogenetic Analysis of Tung Tree Accessions

**Figure 8:**
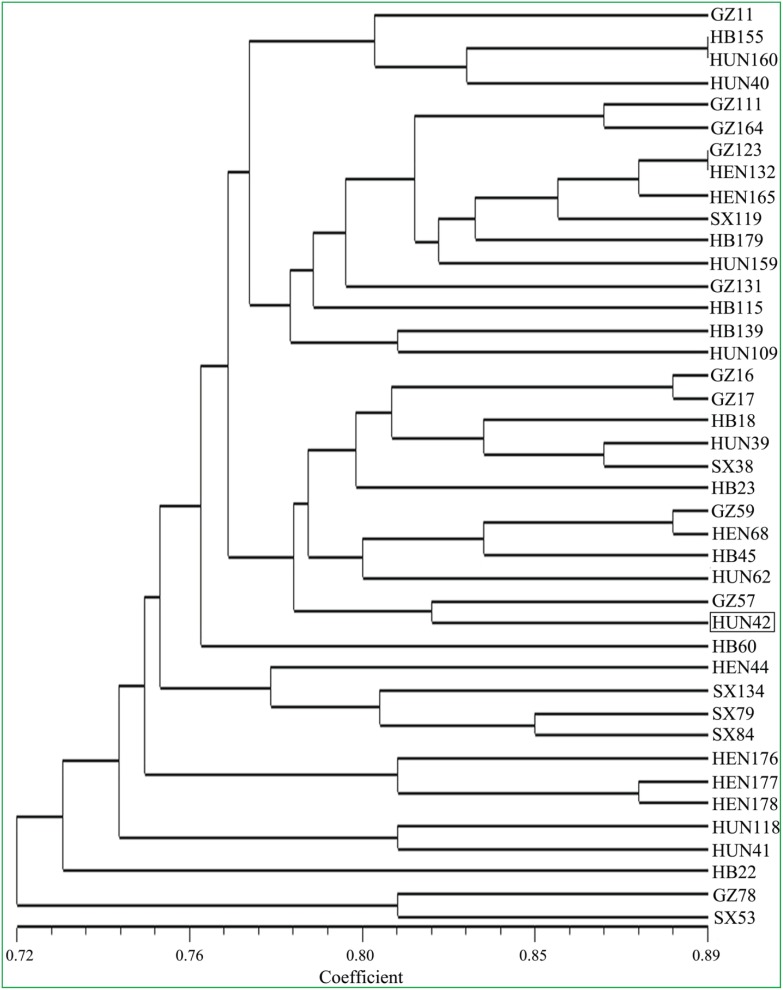
UPGMA dendrogram of the genetic relationships among 41 *V. fordii* accessions. The dendrogram was generated using the Jaccard’s similarity coefficient based on 32 polymorphic SSR-associated genes including 15 new genes identified in this study and 17 genes confirmed based on a previous publication [31]. The boxed “HUN42” was used for cDNA library construction.

Phylogenetic analysis was performed using 32 polymorphic SSR-associated genes including 15 genes identified above and 17 genes confirmed in this study based on a previous publication [31]. Phylogenetic relationships among the 41 *V. fordii* accessions were assessed by constructing an UPGMA dendrogram using similarity coefficients (Figure 8). The similarity values between the tung tree accessions ranged from 0.64 (between HB139 and GZ57) to 0.89 (between GZ123 and HEN132, GZ123 and HEN165, HB155 and HUN160) (data not shown). The dendrogram shows a mixed picture. Although most accessions from the same geographical location were clustered together, a number of exceptions were present in these 41 tung tree accessions (Figure 8). For instance, two accessions HUN42 and HUN160 collected from Hunan Province did not cluster together.

## Discussion

Tung tree is an important oil woody plant due to the widely used tung oil from its seeds. In this report, we described 41 tung tree accessions collected from 5 Chinese Provinces and analyzed the lipid profiles of the seeds. We constructed a cDNA library using tung seed mRNA and sequenced them by Illumina platform-based transcriptome sequencing strategy. We discovered 6,366 SSR motifs with 2–6 nucleotide repeats from 5,404 SSR-containing unique putative transcripts among the 81,805 unigenes. We developed 15 new polymorphic genic-SSR markers in 41 cultivated tung tree accessions. Finally, we confirmed the expression of these 15 genes in developing tung seeds and correlated the expression levels with oil content and fatty acid composition in tung tree seeds.

The economical value of tung tree is due to the unique α-eleostearic acid in tung oil from the seeds. Fatty acid profiles of mature seeds from these tung tree accessions consisted of 7 fatty acids including palmitic acid, stearic acid, oleic acid, linoleic acid, linolenic acid, α-eleostearic acid and β-eleostearic acid. The major fatty acid in tung oil was α-eleostearic acid, which accounted for 77% of the total fatty acids in the seeds. This is in agreement with general observations [4], [12]. The relative abundances of the next 5 fatty acids were 2–8% including linoleic acid, oleic acid, β-eleostearic acid, palmitic acid and stearic acid. The amount of linolenic acid was less than 0.5% and undetectable in oils from several tung tree accessions. During tung tree seed development, the relative ratio of α-eleostearic acid was increasing continuously with a parallel trend to the amount of tung oil accumulation in different stages of the seeds while the ratios of other fatty acids were decreasing. These trends of fatty acid profiles reflect the fact that tung oil is the predominant storage component in tung tree seeds. However, the biological significance of tung oil accumulation in the seeds is not clear whether it is related to insect/pathogen resistance and/or affects seed germination.

MIcroSAtellite software discovered approximately 6.6% of the 81,805 unigenes in tung seeds contained at least one of the considered SSR motifs. This percentage is in agreement with previous studies using EST databases, which shows approximately 3–7% of expressed sequences containing putative SSR motifs [41], [51]. Most of the microsatellites in tung trees were di- and tri-nucleotide. Genomic SSRs identified in some plants such as *C. pepo* and *C. moschata* contained the same predominant di- and tri-nucleotide unit types [52], [53]. The most abundant SSR motifs in tung tree identified in this study were AG/CT and AAG/CTT. A similar bias towards AG and AAG and against CG repeats has been reported in EST-SSRs of many plants including *V. fordii*, *C. pepo* and *A. hypogaea* [30], [37], [52]. Gonzalez-Ibeas *et al*. proposed that this may be due to the tendency of CpG sequences to be methylated which might inhibit transcription [54].

We developed 15 new polymorphic genic-SSR markers in 41 cultivated tung tree accessions. All SSR motifs in the 15 SSR markers contained 20 or more nucleotides. These markers were different from those identified previously in *V. fordii* based on EST sequences and genomic DNA, although the genetic diversity parameters were within a similar range among these studies [30], [31]. The genetic similarity-based dendrogram revealed that most of the 41 accessions from the same geographic region were mainly in the same cluster. Our finding is in agreement with a previous report on genetic diversity of *V. Montana* [30] and *V. fordii* using ISSR markers [55]. One of the reasons for this phenomenon is that the accessions clustering together might have originated from the same geographic region and then were planted in different regions.

The polymorphic genic-SSR markers identified here differ from previously reported tung tree SSR markers in another important way because some of the new SSR markers potentially encoded functional genes. Genbank database search identified 10 of the 15 loci putatively coding for RNA splicing protein mrs2, transcription factor VIP1-like protein, phosphate-induced protein, anthocyanidin reductase, V-type proton ATPase subunit H-like protein, 3′-N-debenzoyl-2′-deoxytaxol N-benzoyltransferase, cadmium resistance 10-like isoform 1, NifU-like protein 4, disease resistance protein RPM1 and protein binding protein. These genes were expressed in developing tung seeds. The expression levels of some of the identified genes were well-correlated with fatty acid composition. However, these genes are not directly related to fatty acid biosynthesis in the seeds. Therefore, it was not surprising that there was a lack of positive correlation between the mRNA levels of these genes and tung oil content in the seeds. Nevertheless, these results demonstrate that genic-SSR markers have special features in comparison with genomic SSR markers, because genic-SSR markers are associated with functional genes and may increase the efficiency of marker-assisted selection [38].

## Conclusions

We reported 41 accessions of tung tree (*Vernicia fordii*) collected from 5 Chinese Provinces and analyzed the lipid profiles of the seeds. A total of 81,805 unigenes were identified by transcriptome sequencing in developing seeds, of which 5,404 SSR-containing loci were identified. Out of 98 loci tested, 15 polymorphic genic-SSR markers were developed and characterized. These genes were expressed in developing tung tree seeds. Ten of the 15 loci putatively coded for functional proteins. These molecular markers increase current SSR marker resources and will greatly benefit future studies on genetic diversity, qualitative and quantitative trait mapping and marker-assisted selection studies in tung tree. The lipid profiles in the seeds of 41 tung tree accessions will be valuable for biochemical and breeding studies.

## Supporting Information

Figure S1 **RNA quality assessment by Agilent 2100 Bioanalyzer.** RNA isolated from seeds at 120 days after flowering (lipid synthesis peak phase) is shown. The quality of RNA isolated from 60 and 165 days after flowering (lipid synthesis initiation phase and ending phase, respectively) were similar (data not shown).
(PDF)

Table S1 **PCR primers used to confirm 17 polymorphic SSR-associated genes in tung tree (** ***V. fordii*** **).** (Microsoft Excel).
(XLSX)

Table S2 **The complete list of 6,366 SSRs from 5,404 unigenes with di-, tri-, tetra-, penta- or hexa-nucleotide repeats in tung tree (** ***V. fordii*** **).** (Microsoft Excel).
(XLSX)

Table S3 **The complete list of the frequency of identified SSR motifs in tung tree (** ***V. fordii*** **).** (Microsoft Excel).
(XLSX)
