# Supplementary material for: Fatty Acid Profile and Unigene-Derived Simple Sequence Repeat Markers in Tung Tree (Vernicia fordii)
Source: PLoS One. 2014 Aug 28;9(8):e105298. doi: 10.1371/journal.pone.0105298 (PMC4148264; doi:10.1371/journal.pone.0105298)
Supplement: Figure S1 — RNA quality assessment by Agilent 2100 Bioanalyzer. RNA isolated from seeds at 120 days after flowering (lipid synthesis peak phase) is shown. The quality of RNA isolated from 60 and 165 days after flowering (lipid synthesis initiation phase and ending phase, respectively) were similar (data not shown). (PDF) [file pone.0105298.s001.pdf]

Fatty Acid Profile and Unigene-derived Simple Sequence Repeat Markers in Tung Tree (*Vernicia fordii*)

Lin Zhang[1,2], Baoguang Jia[1], Xiaofeng Tan[1]*, Chandra S. Thammina[2], Hongxu Long[1], Min Liu[1], Shanna Wen[1], Xianliang Song[3,4], Heping Cao[3]*

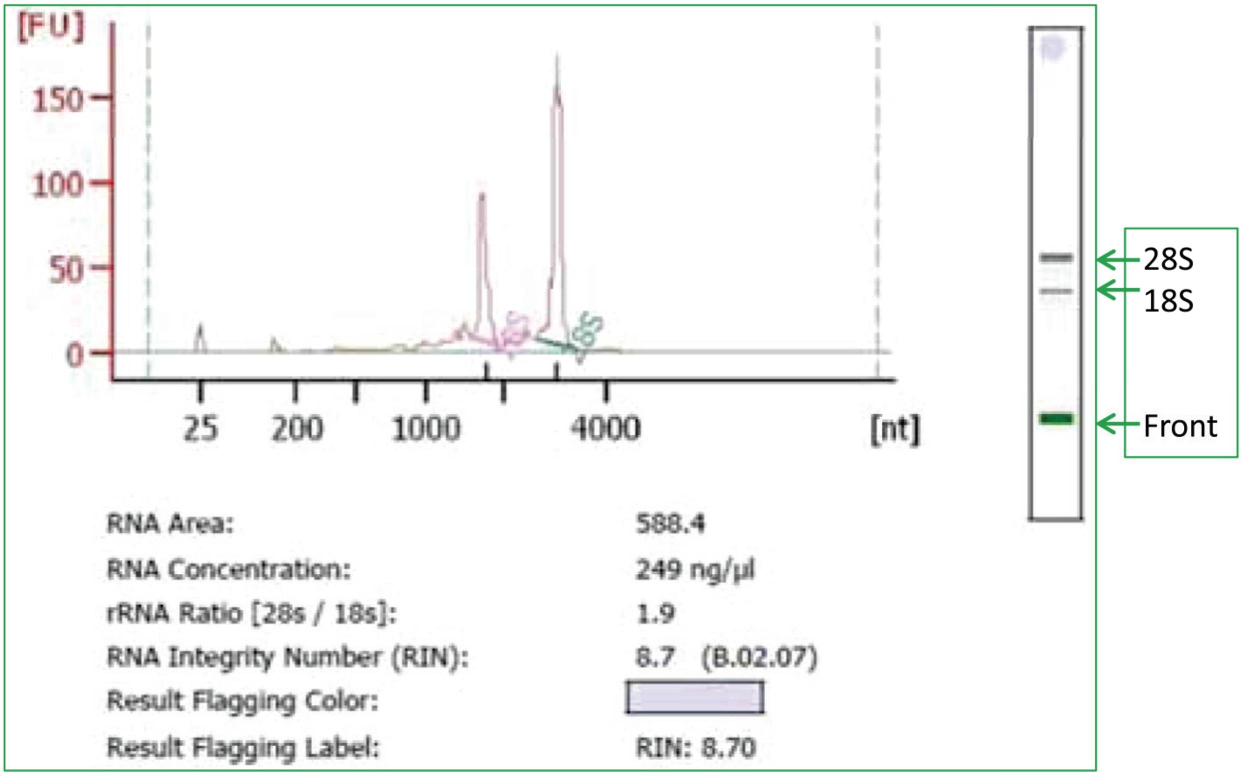

Figure S1. RNA quality assessment by Agilent 2100 Bioanalyzer. RNA isolated from seeds at 120 days after flowering (lipid synthesis peak phase) is shown. The quality of RNA isolated from 60 and 165 days after flowering (lipid synthesis initiation phase and ending phase, respectively) were similar (data not shown).
